# One‐Step Low‐Temperature Synthesis of Metastable *ε*‐Iron Carbide Nanoparticles with Unique Catalytic Properties Beyond Conventional Iron Catalysts

**DOI:** 10.1002/smll.202412217

**Published:** 2025-04-24

**Authors:** Yuma Hirayama, Akira Miura, Motoaki Hirayama, Hiroyuki Nakamura, Koji Fujita, Hiroshi Kageyama, Sho Yamaguchi, Tomoo Mizugaki, Takato Mitsudome

**Affiliations:** ^1^ Department of Materials Engineering Science Graduate School of Engineering Science Osaka University 1‐3 Machikaneyama Toyonaka Osaka 560‐8531 Japan; ^2^ Division of Applied Chemistry Faculty of Engineering Hokkaido University Kita 13 Sapporo Hokkaido 060‐8628 Japan; ^3^ PRESTO Japan Science and Technology Agency (JST) 4‐1‐8 Honcho Kawaguchi Saitama 333‐0012 Japan; ^4^ Department of Applied Physics The University of Tokyo 7‐3‐1 Hongo Bunkyo‐ku Tokyo 113‐8656 Japan; ^5^ RIKEN Center for Emergent Matter Science (CEMS) 2‐1 Hirosawa Wako Saitama 351‐0198 Japan; ^6^ Department of Materials Science and Engineering Graduate School of Engineering Kyoto University Sakyo‐ku Kyoto 606‐8501 Japan; ^7^ Department of Material Chemistry Graduate School of Engineering Kyoto University Nishikyo‐ku Kyoto 615‐8510 Japan; ^8^ Department of Energy and Hydrocarbon Chemistry Graduate School of Engineering Kyoto University Nishikyo‐ku Kyoto 615‐8510 Japan; ^9^ Innovative Catalysis Science Division Institute for Open and Transdisciplinary Research Initiatives (ICS‐OTRI) Osaka University Suita Osaka 565‐0871 Japan; ^10^ Research Center for Solar Energy Chemistry Graduate School of Engineering Science Osaka University 1‐3 Machikaneyama Toyonaka Osaka 560‐8531 Japan

**Keywords:** amination, heterogeneous catalyst, metastable, ε‐iron carbide

## Abstract

*ε*‐Iron carbide has garnered increasing interest for its superior magnetic characteristics and catalytic performance compared to other iron carbides. However, its metastable nature has posed significant challenges for synthesis, often requiring ultrahigh pressure, multistep processes, complex reaction condition control, and highly toxic reagents. Consequently, the properties of *ε*‐iron carbide remain largely unexplored. A simplified synthesis method for *ε*‐iron carbide can accelerate the exploration of new functionalities. In this study, a novel one‐step selective synthesis method for *ε*‐iron carbide nanoparticles under mild conditions via a wet‐chemical approach is presented. In this method, Fe_3_(CO)_12_, cetyltrimethylammonium bromide (CTAB), and bis(pinacolato)diboron (B_2_pin_2_) are added to hexadecylamine and reacted at 220 °C—a simple process that eliminates the need for extreme pressures and toxic substances. Detailed investigations elucidate the crucial roles of CTAB and B_2_pin_2_ in facilitating the selective formation of *ε*‐iron carbide. This accessible and efficient synthesis process for *ε*‐iron carbide can further enable the discovery of unprecedented catalytic properties in the reductive amination of benzaldehyde, distinct from those of conventional iron nanoparticle catalysts. Density functional theory calculations reveal insights into the electronic states responsible for the distinct activity of the *ε*‐iron carbide nanoparticles.

## Graphical abstract

A novel one‐step method enables the selective synthesis of *ε*‐iron carbide nanoparticles under mild conditions. This accessible approach uncovers unprecedented catalytic properties of *ε*‐iron carbide in reductive amination, advancing the exploration of *ε*‐iron carbide's unique functionalities.

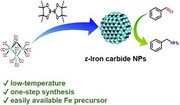

## 1 Introduction

Iron carbides, including Fe_3_C, Fe_5_C_2_, Fe_7_C_3_, and *ε*‐iron carbide (*ε*‐Fe_x_C; x = 2–3), have been extensively studied owing to their critical roles in industrial and technological applications. These materials exhibit high mechanical strengths, chemical stability, particularly against corrosion, and magnetic properties, which are essential for applications in metallic alloys and hard coatings.^[^ ^1^, ^2^, ^3^, ^4^ ^]^ Recent advances in materials science have also expanded their applications to emerging fields, such as biomedicines, magnetic storage devices, and catalysts, reflecting their potential for diverse functionality.^[^ ^5^, ^6^, ^7^, ^8^, ^9^, ^10^, ^11^ ^]^ Among these carbide phases, *ε*‐iron carbide has attracted significant attention for its high saturation magnetization, which enables applications in bioimaging, drug delivery, and magnetic hyperthermia.^[^ ^12^, ^13^, ^14^, ^15^ ^]^ Moreover, *ε*‐iron carbide has demonstrated high catalytic activity for the Fischer–Tropsch synthesis of hydrocarbons, as well as for the hydrogenation of oxalate esters,^[^ ^16^, ^17^, ^18^, ^19^, ^20^, ^21^ ^]^ making it a promising material for both magnetic and catalytic applications. However, *ε*‐iron carbide is a metastable phase that forms only under ultrahigh‐pressure conditions exceeding 13 GPa,^[^ ^22^ ^]^ presenting a significant barrier to its synthesis and broader application.

**Figure 1:**
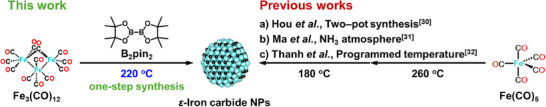
Comparison of low‐temperature synthesis methods for *ε*‐iron carbide nanoparticles (NPs) using wet‐chemical methods: the method developed in this study versus conventional multistep processes.

To address these challenges, recent studies have explored alternative methods for the facile synthesis of *ε*‐iron carbide, including thermal decomposition methods,^[^ ^23^, ^24^, ^25^, ^26^ ^]^ gas–solid reaction methods,^[^ ^19^, ^20^, ^27^, ^28^, ^29^ ^]^ and wet‐chemical methods.^[^ ^30^, ^31^, ^32^ ^]^ These approaches have successfully mitigated the extreme synthesis conditions traditionally required for *ε*‐iron carbide formation. However, each approach has specific limitations that restrict its broader application. For instance, thermal decomposition methods still demand high temperatures exceeding 600 °C, and gas–solid reaction methods often involve specialized equipment and demand complex temperature and pressure control. Wet‐chemical techniques, in comparison, operate at significantly lower temperatures (260–350 °C) and are less reliant on specialized equipment, offering an accessible route for *ε*‐iron carbide synthesis. For instance, Hou et al. successfully synthesized *ε*‐iron carbide via a two‐step process involving the thermal decomposition of Fe(CO)_5_ to form Fe@Fe_3_O, followed by heating at 260 °C (**Figure** **1**a).^[^ ^30^ ^]^ Ma et al. employed a similar approach, using an NH₃ atmosphere to produce *ε*‐iron carbide in two distinct stages (Figure 1b).^[^ ^31^ ^]^ Thanh et al. thermally decomposed Fe(CO)_5_ in the presence of a long‐chain amine using temperature‐programmed methods (Figure 1c).^[^ ^32^ ^]^ While these wet‐chemical approaches demonstrate the feasibility of synthesizing *ε*‐iron carbide under relatively mild conditions, these procedures require multiple steps, such as the separation and purification of the intermediate compounds. Additionally, these approaches rely on the use of Fe(CO)_5_, a hazardous precursor that poses safety and handling challenges due to factors such as its strict exposure limit (0.1 ppm) and spontaneous flammability. Thus, despite recent advances in synthesis techniques, the development of a more accessible and simpler method for synthesizing *ε*‐iron carbide remains a significant challenge.

Herein, we report a one‐step low‐temperature synthesis method for *ε*‐iron carbide nanoparticles (NPs) using the nonvolatile and easily available Fe_3_(CO)_12_, cetyltrimethylammonium bromide (CTAB), and bis(pinacolato)diboron (B_2_pin_2_). This method avoids the multistep complexities and hazards of conventional approaches. Detailed investigations into the roles of CTAB and B_2_pin_2_ revealed their contributions to selective *ε*‐iron carbide formation. Furthermore, the unique catalytic properties of *ε*‐iron carbide, distinct from those of conventional iron NP catalysts, were demonstrated in the reductive amination of benzaldehyde, with density functional theory (DFT) calculations revealing insights into the electronic states of *ε*‐iron carbide responsible for its high activity.

## 2 Results and Discussion

**Figure 2:**
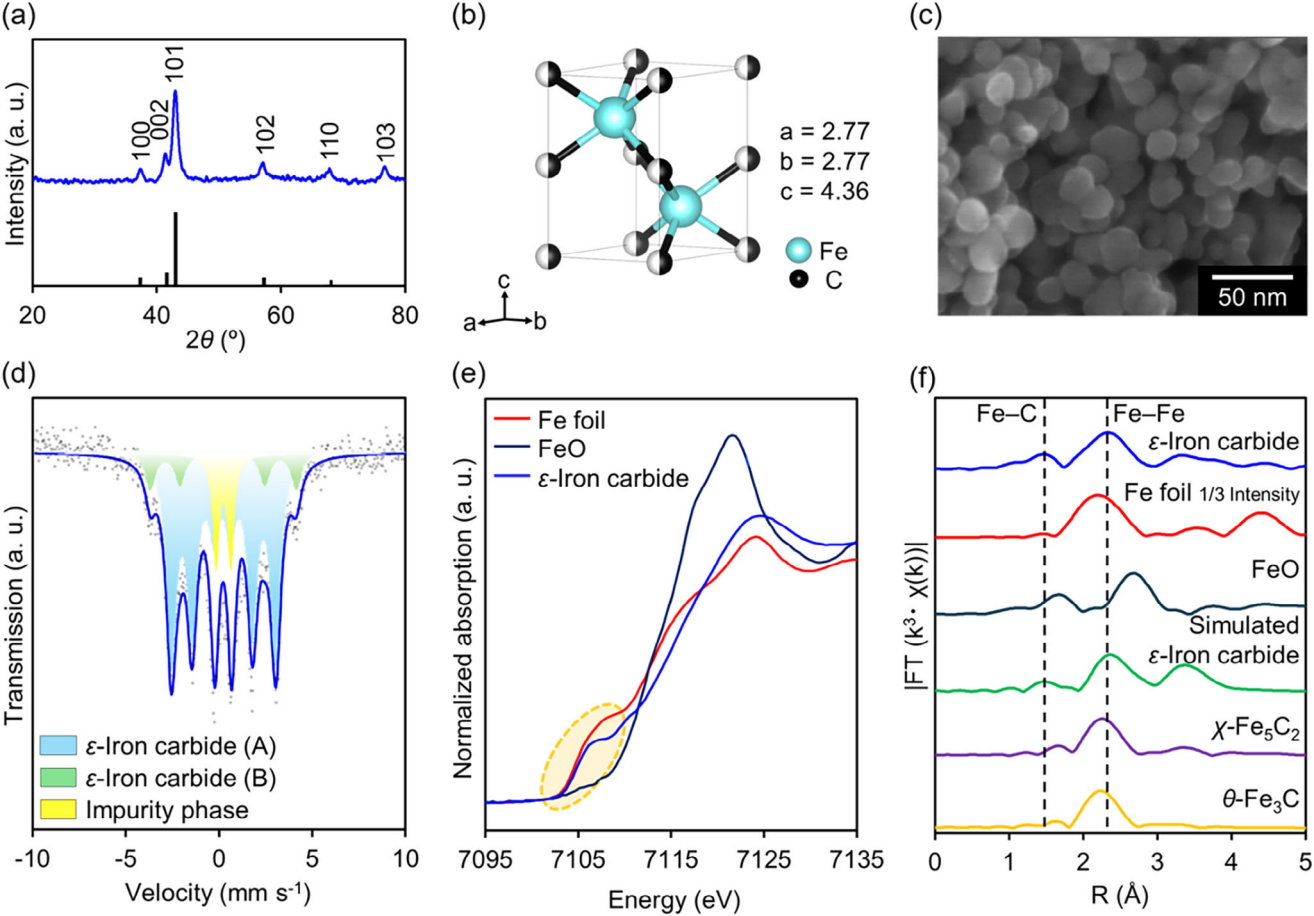
a) XRD pattern of the obtained product (black bars below the pattern show the diffraction peaks referring to JCPDS card number 36–1249). b) Crystal structure of *ε*‐iron carbide. c) SEM image of *ε*‐iron carbide. d) ^57^Fe Mössbauer spectrum of *ε*‐iron carbide NPs. e) Fe *K*‐edge XANES spectra of Fe foil, FeO, and *ε*‐iron carbide. f) Fourier transformations of *k* ^3^‐weighted Fe *K*‐edge EXAFS spectra of *ε*‐iron carbide, Fe foil, FeO, and simulated *ε*‐iron carbide, *χ*‐Fe_5_C_2_, and *θ*‐Fe_3_C.

The typical synthesis procedure for *ε*‐iron carbide is as follows. Fe_3_(CO)_12_ (0.33 mmol), CTAB (0.06 mmol), and B_2_pin_2_ (2 mmol) were added to hexadecylamine at 25 °C under an Ar atmosphere. The mixture was then heated to 220 °C and held at that temperature for 18 h. After cooling, the resulting solid was collected by a magnet and washed with *n*‐hexane followed by chloroform, yielding *ε*‐iron carbide as a black solid. **Figure** **2**a presents the X‐ray diffraction (XRD) pattern of the obtained product, which corresponds to that of *ε*‐iron carbide (JCPDS no. 36–1249). No peaks were detected for metallic iron, iron oxides, other iron carbide compositions, or graphite, indicating selective synthesis of *ε*‐iron carbide. Rietveld refinement using synchrotron XRD data showed that the lattice parameters of *ε*‐iron carbide were a = 2.77005(9) and c = 4.3557(2) (Figure 2b; Figure S1 and Tables S1 and S2, Supporting Information), which are very close to the reported values for *ε*‐Fe_2_C.^[^ ^17^ ^]^ Figure 2c shows the representative Scanning Electron Microscopy (SEM) image of the obtained *ε*‐iron carbide, revealing spherical NPs with a mean diameter of 28 nm (Figures S2,S3, Supporting Information for detailed characterization). ^57^Fe Mössbauer spectroscopy was used to identify and quantify the iron phases (Figure 2d): the two sets of sextets with hyperfine fields of 17.4 and 24.3 T are assigned to *ε*‐iron carbide, in close agreement with previous reports^[^ ^17^ ^]^ in addition to a paramagnetic doublet with a quadruple splitting of 0.9 mm s^−1^, which is most likely an impurity contribution. Based on their area intensities, *ε*‐iron carbide was estimated to be 86% (Table S3, Supporting Information). Figure 2e shows the X‐ray absorption near‐edge structure (XANES) spectra of the *ε*‐iron carbide along with those of Fe foil and FeO. The absorption edge energy of *ε*‐iron carbide was considerably lower than that of FeO, and very close to that of Fe foil, suggesting that the Fe species in the *ε*‐iron carbide exist in a metal‐like oxidation state. Furthermore, the Fourier transform of the extended XAFS (FT‐EXAFS) spectrum of the *ε*‐iron carbide revealed two peaks at 1.5 and 2.3 Å, assigned to the Fe─C and Fe─Fe bonds, respectively (Figure 2f); the peak positions aligned closely with those of simulated *ε*‐iron carbide and deviated slightly from those of Fe foil, as well as from those of simulated *χ*‐Fe_5_C_2_ and *θ*‐Fe_3_C, confirming the successful formation of *ε*‐iron carbide. To determine the local structures of the Fe species in the *ε*‐iron carbide, curve fitting was conducted (Figure S4 and Table S4, Supporting Information), which revealed that the coordination numbers (CN) of the nearest Fe─C and Fe─Fe bonds in the *ε*‐iron carbide were 3.0 and 9.9, respectively. Notably, the Fe─C to Fe─Fe coordination number ratio (3.3) is considerably smaller than the ideal value (4.0). This result indicates that nano‐sized *ε*‐iron carbide with coordinatively unsaturated Fe species was successfully synthesized.

**Figure 3:**
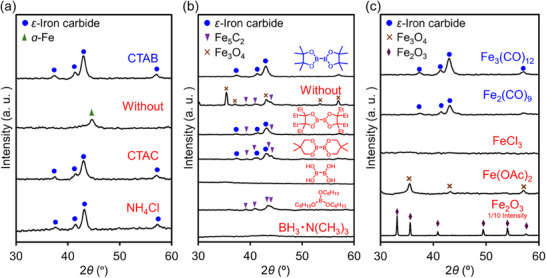
XRD patterns of samples synthesized with various a) halides, b) boron compounds, and c) iron precursors.

Following the successful synthesis of *ε*‐iron carbide, we investigated the influence of CTAB, B_2_pin_2_, and Fe_3_(CO)_12_ on the synthesis parameters by systematically varying the halogen sources, boron compounds, and iron precursors (**Figure** **3**). Figure 3a shows the effect of halogen sources. *ε*‐Iron carbide was successfully synthesized using cetyltrimethylammonium chloride (CTAC) or NH_4_Cl instead of CTAB, whereas poorly crystalline *α*‐Fe was obtained in the absence of halides. This result is consistent with previous reports that halides enhance the crystallinity of iron seeds formed from the decomposition of iron carbonyls through strong interactions between the iron seeds and halides, after which the crystallized iron undergoes carburization to form iron carbide.^[^ ^33^, ^34^, ^35^ ^]^ Given these findings, we selected CTAB as a halogen source for subsequent experiments due to its ease of handling, particularly its no‐hygroscopic nature. Figure 3b illustrates the influence of boron compounds. When B_2_Epin_2_ or B_2_nep_2_, both structurally similar to B_2_pin_2_, were used, *ε*‐iron carbide was formed along with Fe_5_C_2_ (Figure S5, Supporting Information for detailed characterization). In contrast, the use of other boron compounds such as tetrahydroxydiboron, trihexylborate, and borane trimethylamine complex did not afford *ε*‐iron carbide. These results suggest that B_2_pin_2_ is the most effective boron compound for the selective formation of *ε*‐iron carbide. Figure 3c presents the effects of various iron precursors. *ε*‐Iron carbide was formed exclusively upon using Fe_3_(CO)_12_ and Fe_2_(CO)_9_, while using FeCl_3_, Fe(OAc)_2_, and Fe_2_O_3_ did not lead to *ε*‐iron carbide formation; the XRD spectrum of the use of FeCl_3_ showed no discernible peaks, indicating the formation of an amorphous material, Fe(OAc)_2_ resulted in Fe_3_O_4_ formation, and Fe_2_O_3_ remained unchanged throughout the reaction. These findings highlight the importance of iron carbonyls in enabling *ε*‐iron carbide synthesis.

**Figure 4:**
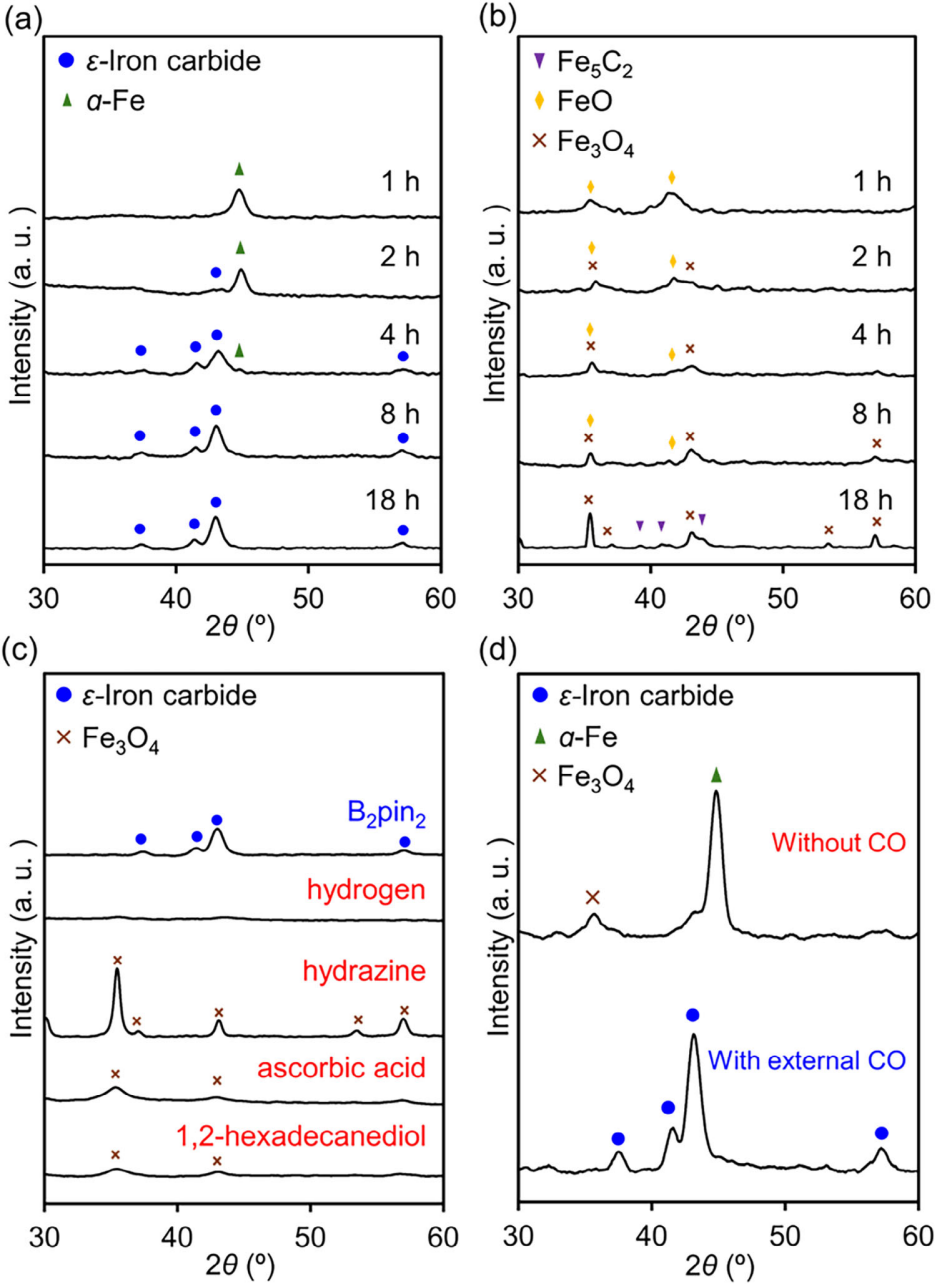
Structural evolutions during the synthesis of *ε*‐iron carbide a) with and b) without B_2_pin_2_. Synthesis conditions: Fe_3_(CO)_12_ (0.33 mmol), hexadecylamine (20 mmol), CTAB (0.06 mmol), B_2_pin_2_ (2.0 mmol), 220 °C. c) XRD patterns of samples synthesized with various reductants. d) Effect of CO removal on *α*‐Fe transformation: After generating *α*‐Fe, CO was removed, followed by either continued heating or reintroduction of CO at 220 °C for 18 h.

Among the parameters for *ε*‐iron carbide synthesis, the use of B_2_pin_2_ as an additive is particularly noteworthy, as its application in the synthesis of *ε*‐iron carbide has not been reported thus far. Therefore, we further investigated the role of B_2_pin_2_ in facilitating the formation of *ε*‐iron carbide. The time‐dependent evolution of XRD patterns during *ε*‐iron carbide synthesis with and without B_2_pin_2_ was studied (**Figure** **4**a). After 1 h of synthesis with B_2_pin_2_, the diffraction peak corresponding to *α*‐Fe appeared at ≈44.7°. An increase in the reaction time from 2 to 4 h weakened the *α*‐Fe peak and enhanced the *ε*‐iron carbide peaks. This observation suggests that the *ε*‐iron carbide formation process involved the initial decomposition of Fe_3_(CO)_12_ into *α*‐Fe, followed by the carburization of *α*‐Fe to *ε*‐iron carbide (see Figure S6, Supporting Information for structural evolution). In contrast, in the synthesis without B_2_pin_2_, the diffraction peaks assigned to FeO were observed after 1 h, which gradually transformed into those of Fe_3_O_4_ over time (Figure 4b). These results demonstrate the crucial role of B_2_pin_2_ in the synthesis of *ε*‐iron carbide, as it facilitates carburization while preventing the oxidation of Fe formed from the decomposition of Fe_3_(CO)_12_ into FeO_x_. Based on the finding that B_2_pin_2_ acts as a reductant, we attempted the synthesis of *ε*‐iron carbide using various other reducing agents (Figure 4c). Notably, only B_2_pin_2_ was able to effectively promote the selective formation of *ε*‐iron carbide, whereas other reducing agents did not lead to *ε*‐iron carbide formation under similar conditions. This confirms the indispensable role of B_2_pin_2_ as a reductant in *ε*‐iron carbide synthesis. The role of B_2_pin_2_ as a reducing agent has been recently reported for the reduction of metal oxide surfaces.^[^ ^36^, ^37^ ^]^ Furthermore, to gain insights into the carbon source for *ε*‐iron carbide formation, we conducted control experiments (Figure 4d). After heating at 220 °C for 1 h to generate *α*‐Fe, followed by the removal of CO and further heating at 220 °C, no change was observed in the *α*‐Fe. In contrast, when CO gas was introduced after CO removal, *ε*‐iron carbide was formed upon heating. These results clearly demonstrate that CO serves as the carbon source for *ε*‐iron carbide formation.

**Figure 5:**
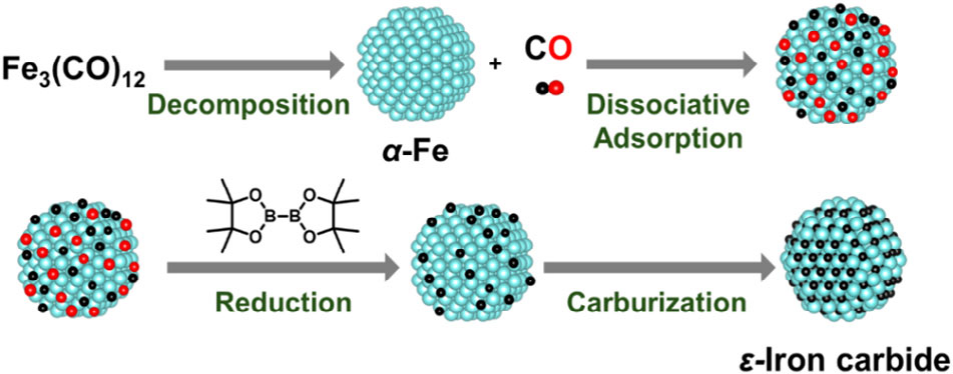
A proposed formation path for *ε*‐iron carbide NPs.

Based on the above control experiments, we propose a mechanism for *ε*‐iron carbide formation, as shown in **Figure** **5**. First, Fe_3_(CO)_12_ decomposes upon heating, generating *α*‐Fe NPs and CO gas. The CO gas then dissociatively adsorbs onto the surfaces of the *α*‐Fe NPs, splitting into carbon atoms and oxygen atoms. Subsequently, B_2_pin_2_ acts as a reducing agent, removing the surface oxygen species, which facilitates the carburization of the *α*‐Fe NPs by the surface carbon, ultimately leading to the formation of *ε*‐iron carbide. The dissociative adsorption of CO onto Fe is well established in Fischer–Tropsch synthesis.^[^ ^38^, ^39^, ^40^, ^41^ ^]^

**Figure 6:**
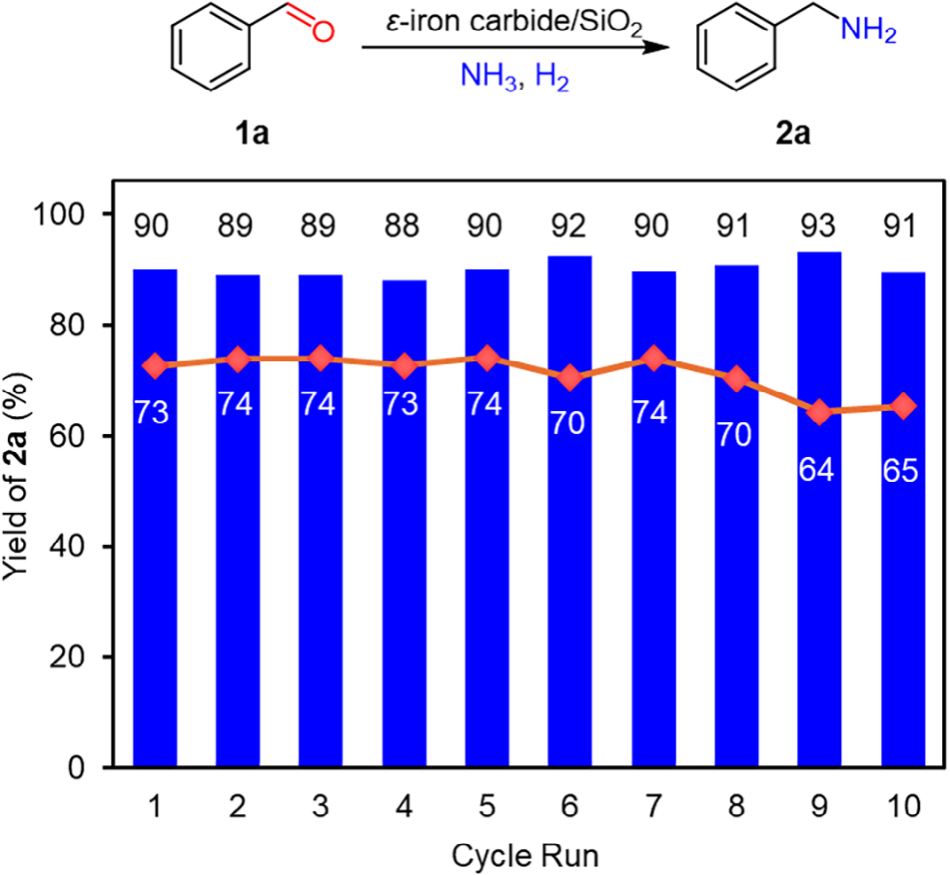
Reuse experiments. Reaction conditions: *ε*‐iron carbide/SiO_2_ (0.1 g), **1a** (0.5 mmol), ethanol (3 mL), H_2_ (3.7 MPa), NH_3_ (0.3 MPa), 150 °C. Reaction time: 3 h (blue columns), 50 min (red diamonds).

The catalytic property of the *ε*‐iron carbide NPs was investigated in the reductive amination of benzaldehyde (**1a**) using ammonia as the amine source because amination is one of the most important and industrially reliable methods for synthesizing primary amines. There have been few reports on amination using iron catalysts,^[^ ^42^, ^43^, ^44^, ^45^ ^]^ highlighting the significant challenge in developing iron‐based catalysts for this reaction. **Table** **1** shows the results of the catalytic test comparing *ε*‐iron carbide NPs (unsupported), *ε*‐iron carbide/SiO_2_, Fe_5_C_2_/SiO_2_ (Figure S7, Supporting Information for Fe_5_C_2_ characterization), Fe/SiO_2_ and FeO_x_/SiO_2_. Notably, *ε*‐iron carbide (unsupported) exhibited remarkably high catalytic activity in the reductive amination of **1a**, achieving an 89% yield of benzylamine (**2a**) comparable to that of *ε*‐iron carbide/SiO₂ (90% yield). In contrast, Fe_5_C_2_/SiO_2,_ a commonly available iron carbide, exhibited much lower activity with a 32% yield. Meanwhile, Fe/SiO_2_ and FeO_x_/SiO_2_ showed negligible activity for the formation of **2a** and predominantly afforded benzylideneamine. These results demonstrate the distinct and superior activity of *ε*‐iron carbide (see Figure S8, Supporting Information for time‐course and Figure S9 (Supporting Information) for proposed reaction path). The durability of *ε*‐iron carbide/SiO_2_ was also investigated through recycling experiments. It is noted that the activity of *ε*‐iron carbide/SiO_2_ was maintained even in the nineth recycling (**Figure** **6**). ^57^Fe Mössbauer, XRD, XANES, and TEM analysis of the used *ε*‐iron carbide/SiO_2_ revealed no significant structural and morphology changes (Figures S10–S13, Supporting Information), confirming the stability of *ε*‐iron carbide under the reaction conditions. This represents the first demonstration of the catalytic performance of *ε*‐iron carbide in liquid‐phase molecular transformations. The high catalytic performance of *ε*‐iron carbide can be attributed to the presence of coordinatively unsaturated Fe sites, as observed in the EXAFS analysis. Such findings significantly expand the potential of *ε*‐iron carbide as a novel class of iron‐based catalysts.

**Table 1:** Reductive amination of benzaldehyde (**1a**) to benzylamine (**2a**) using Fe‐based catalysts.

Entry	Catalyst	Conversion of 1a [%]	Yield of 2a [%]
1	*ε*‐iron carbide NPs	>99	89
2	*ε*‐iron carbide/SiO_2_	>99	90
3	Fe_5_C_2_/SiO_2_	>99	32
4	Fe/SiO_2_	>99	<1
5	FeO_x_/SiO_2_	>99	<1

Reaction conditions: Fe catalyst (Fe: 10 mol%), **1a** (0.5 mmol), ethanol (3 mL), NH_3_ (0.3 MPa), H_2_ (3.7 MPa), 150 °C, 3 h. Conversion and yield were determined by gas chromatography using the internal standard technique.

**Figure 7:**
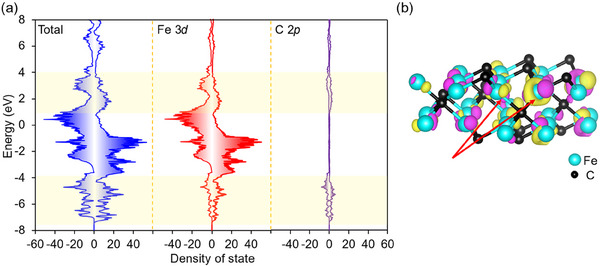
a) Total density of states and projected density of states (PDOS) of Fe and C atom in *ε*‐iron carbide. b) Visualization of wavefunctions near the Fermi energy for *ε*‐iron carbide. The light yellow and purple isosurfaces represent the positive and negative phases of wavefunctions respectively.

Lastly, to gain key insights into the structure‐activity relationship, DFT calculations were performed to investigate the electronic states of *ε*‐iron carbide. The density of states (DOS) and projected density of states (PDOS) are presented in **Figure** **7**a. The PDOS for 2*p* orbitals of carbon atoms shows contributions from occupied states in the −7.5 to −4.0 eV range and unoccupied states in the 1.0 to 4.0 eV region. The PDOS for 3*d* orbitals of iron atoms exhibits significant overlap with the carbon PDOS in the same energy regions, suggesting the presence of covalent Fe─C bonding. Moreover, a high density of states is observed near the Fermi energy, indicating a substantial electronic population in this region. Figure 7b presents a visualization of the wavefunctions near the Fermi level of *ε*‐iron carbide. These wavefunctions exhibit a spatial distribution that deviates from alignment along the Fe─C bond axis, confirming the presence of localized *d*‐electrons of iron near the Fermi level that do not participate in covalent Fe─C bonding. These localized *d*‐electrons are expected to interact strongly with molecular hydrogen, potentially facilitating the reductive amination.

## 3 Conclusion

In this study, we successfully synthesized the metastable compound *ε*‐iron carbide under mild conditions. Unlike traditional methods, which require extremely high‐pressure conditions and specialized equipment, our approach enables the one‐step selective synthesis of nano‐sized *ε*‐iron carbide at ambient pressure without the use of highly toxic reagents such as CO or Fe(CO)_5_. Our experimental results highlighted the crucial roles of CTAB and B_2_pin_2_ in the formation of *ε*‐iron carbide, with B_2_pin_2_ identified as an effective and unique reducing agent in the synthesis process. This simplified and accessible method offers a promising pathway for the efficient production of *ε*‐iron carbide. Furthermore, the obtained *ε*‐iron carbide NPs exhibited distinct catalytic activity in the reductive amination of benzaldehyde, while conventional iron NPs showed almost no activity, demonstrating the utility of *ε*‐iron carbide as a novel iron‐based catalyst for diverse organic syntheses. We believe that this advancement will make *ε*‐iron carbide more readily available for further investigation, enabling broader exploration and expanding its potential applications, which could contribute to future innovations in materials science.

## 4 Experimental Section

### Materials

All commercially available chemicals were used as received. Fe_3_(CO)_12_ (1–10% methylalcohol), Fe_2_(CO)_9_ (>98%), and borane trimethylamine complex (>97%) were purchased from Sigma–Aldrich (St. Louis, U.S.A). Hexadecylamine (>95%), bis(pinacolato)diboron (B_2_pin_2_; > 99%), hexadecyltrimethylammonium chloride (CTAC; > 95%), 4,4,4′,4′,5,5,5′,5′‐octaethyl‐2,2′‐bi(1,3,2‐dioxaborolane) (B_2_Epin_2_; > 98%), bis(neopentyl glycolato)diboron (B_2_nep_2_; > 98%), tetrahydroxydiboron (B_2_(OH)_4_; > 98%), trihexylborate (>98%), iron (II) acetate (Fe(OAc)_2_; > 90%), L‐ascorbic acid (>99%), and 1,2‐hexadecanediol (>98%) were acquired from Tokyo Chemical Industry Co., Ltd. (Tokyo, Japan). Cetyltrimethylammonium bromide (CTAB; > 99%), ammonium chloride (NH_4_Cl; > 99.5%), *α*‐iron(III) oxide (Fe_2_O_3_; > 99.9%), hydrazine monohydrate (H_2_NNH_2_·H_2_O; > 97%), iron (III) nitrate nonahydrate (>99%), benzaldehyde (>98%), biphenyl (>98%), ethanol (>99.5%), chloroform (>99%), and *n*‐hexane (>99%) were obtained from Fujifilm Wako Pure Chemical Corporation (Osaka, Japan). Anhydrous iron (III) chloride was obtained from Kanto Chemical Co., Inc. (Tokyo, Japan). SiO_2_ (CARiACT, Q‐6) was supplied by Fuji Silysia Chemical Ltd. (Aichi, Japan).

### Synthesis of ε‐Iron Carbide NPs

Hexadecylamine (20 mmol) and CTAB (0.06 mmol) were added to a Shlenk flask and stirred for 1 h under vacuum at 120 °C. After cooling to 25 °C, Fe_3_(CO)_12_ (0.33 mmol) and B_2_pin_2_ (2.0 mmol) were added under Ar flow. Subsequently, the temperature was increased to 220 °C at a rate of 5 °C min^−1^ and held constant for 18 h. After cooling the mixture to room temperature, the solid was separated from the liquid phase by a magnet and washed with *n*‐hexane and chloroform to afford the desired *ε*‐iron carbide NPs as a black powder.

### Synthesis of Fe_5_C_2_ NPs

Hexadecylamine (20 mmol) and CTAB (0.06 mmol) were added to a Shlenk flask and stirred for 1 h under vacuum at 120 °C. After cooling to 25 °C, Fe_3_(CO)_12_ (0.33 mmol) was added under Ar flow. Subsequently, the temperature was increased to 320 °C at a rate of 5 °C min^−1^ and held constant for 18 h. After cooling the mixture to room temperature, the solid was separated from the liquid phase by a magnet and washed with *n*‐hexane and chloroform to afford the desired Fe_5_C_2_ NPs as a black powder.

### Synthesis of ε‐Iron Carbide/SiO_2_ and Fe_5_C_2_/SiO_2_

*ε*‐iron carbide NPs or Fe_5_C_2_ NPs (40 mg) were dispersed in chloroform (50 mL) and stirred with SiO_2_ (1.0 g) at 25 °C for 6 h to afford *ε‐*iron carbide/SiO_2_ or Fe_5_C_2_/SiO_2_, respectively.

### Synthesis of Fe NP/SiO_2_ and FeO_x_ NP/SiO_2_

SiO_2_ (1.0 g) was added to a 2 mm ethanolic solution of Fe(NO_3_)_3_ (50 mL) and stirred for 48 h at 25 °C, followed by evaporation at 75 °C. The resulting catalyst precursor was then reduced under an H_2_ gas flow while heating from 25 to 900 °C at a rate of 5 °C min^−1^, followed by holding at 900 °C for 1 h to yield the desired Fe NP/SiO_2_. The FeO_x_ NP/SiO_2_ catalyst was prepared in a similar way, but the heating conditions were changed to 600 °C for 2 h under air.

### Reductive Amination Procedure

The reductive amination of benzaldehyde with ammonia gas was carried out in a 50‐mL stainless steel autoclave equipped with a Teflon vessel. The vessel was charged with benzaldehyde (0.5 mmol), *ε*‐iron carbide/SiO_2_ (0.1 g), ethanol (3 mL), and a Teflon‐coated magnetic stir bar was added. The reaction mixture was stirred vigorously at 150 °C under 3.7 MPa of H_2_ and 0.3 MPa of NH_3_. After the reaction, the reaction solution was analyzed by GC–FID to determine the conversion and yield using biphenyl as an internal standard. In addition, to obtain the benzylamine hydrochloride salt, the crude reaction mixture was filtered to remove the catalyst, and ammonia was removed under vacuum conditions. The mixture was then added to a hydrogen chloride solution (1.25 m in 1,4‐dioxane), and the solvent was removed to give the pure benzylamine hydrochloride salt for NMR analysis.

The yields of primary amine and secondary imine are calculated as follows:

(1) $$\begin{array}{ccc} \mathrm{Yield}\;\left(\%\right)\;\mathrm{of}\;\mathrm{primary}\;\mathrm{amine}\; & = & \frac{\mathrm{the}\;\mathrm{mol}\;\mathrm{of}\;\mathrm{primary}\;\mathrm{amine}\;\mathrm{product}}{\mathrm{the}\;\mathrm{initial}\;\mathrm{mol}\;\mathrm{of}\;\mathrm{substrate}}\;\\ & & \times 100\% \end{array}$$

(2) $$\begin{array}{ccc} \mathrm{Yield}\;\left(\%\right)\;\mathrm{of}\;\mathrm{secondary}\;\mathrm{imine}\; & = & \frac{\mathrm{the}\;\mathrm{mol}\;\mathrm{of}\;\mathrm{secondary}\;\mathrm{imine}\;\mathrm{product}\;}{\mathrm{the}\;\mathrm{initial}\;\mathrm{mol}\;\mathrm{of}\;\mathrm{substrate}}\\ & & \times 100\% \end{array}$$

### Recycling Experiment

After the reaction, *ε*‐iron carbide/SiO_2_ was removed by centrifugation, and the primary amine yield was determined by GC. The spent catalyst was washed with ethanol for the reuse experiments. No other catalyst pretreatment was required.

### Characterization

XRD patterns were obtained using the Philips X'Pert‐MPD (PANanalytical B.V., Almelo, Netherlands) using Cu *Kα* radiation (45 kV, 40 mA). Spectra were smoothed by Savitzky–Golay method. SEM analysis was conducted on the S‐5000H (Hitachi Hi‐tech corporation, Tokyo, Japan) at 20 kV. TEM analysis was carried out with JEM‐2100 (JEOL Ltd., Tokyo, Japan) at 200 kV. The *K*‐edge X‐ray absorption spectra were recorded at the BL01B1 line, using a Si(111) monochromator at the SPring‐8 facility of the Japan Synchrotron Radiation Research Institute (JASRI) (Harima, Japan) with approval of 2024A1548 and 2024B1602. Data analysis was performed using the Demeter ver. 0.9.26 program.^[^ ^46^ ^]^ After normalization at the edge height, the *k* ^3^‐weighted χ spectra were extracted. Subsequently, these spectra in the *k* range of 2–14 Å^−1^ were Fourier‐transformed into the *R*‐space. Curve‐fitting analysis was conducted in the *R*‐range of 1.0–3.0 Å using the back‐scattering amplitudes and phase‐shifting functions of the Fe─Fe and Fe─C bonds, which were calculated using the FEFF6L program.^[^ ^47^ ^]^ Theoretical Radial Distribution Function was calculated using the FEFF6L program and the crystal structures of *ε*‐iron carbide (*ε*‐Fe_2_C),^[^ ^48^ ^]^ *χ*‐Fe_5_C_2_^[^ ^49^ ^]^ and *θ*‐Fe_3_C^[^ ^50^ ^]^ as the input data. Synchrotron XRD was measured at the BL13XU and BL02B2 at SPring‐8 (JASRI) with approval of 2024B1777 and 2024B2419. Rietveld refinement was performed using RIETAN‐FP.^[^ ^51^ ^] 57^Fe Mössbauer spectroscopy was conducted in a transmission geometry at room temperature by employing ^57^Co in metallic Rh as a γ‐ray source. The powder sample was vacuum sealed in multiple layers of polyethylene bags with a thickness of 0.1 mm in order to avoid direct contact with air. The velocity scale was calibrated by a spectrum of *α*‐Fe foil at room temperature. The isomer shift was determined with respect to *α*‐Fe. The Mössbauer spectra were analyzed using MossA.^[^ ^52^ ^]^ Gas chromatography with flame ionization detection (GC‐2014, Shimadzu Corporation, Kyoto, Japan) was performed using an InertCap for amines (GL Sciences, Tokyo, Japan, 30 m × 0.32 mm i.d.) and a packed column (GL Sciences, Tokyo, Japan, PEG‐HT and KOH on Uniport HP, 3.1 m × 3.2 mm i.d.). ^1^H and ^13^C NMR spectra were recorded on a JEOL JNM‐ESC400 (JEOL Ltd., Tokyo, Japan). The ^1^H NMR chemical shifts were reported in parts per million (ppm) using tetramethylsilane (0.00 ppm), while the ^13^C NMR chemical shifts were reported in ppm using dimethyl sulfoxide‐*d*_6_ (DMSO‐*d*_6_) (39.50 ppm). NMR multiplicities were reported using the following abbreviations: s, singlet; d, doublet; m, multiplet; br, broad; *J*, coupling constants in hertz.

### Detail of Ab Initio Calculation

All ab initio calculations were implemented in the Vienna ab initio simulation package (VASP).^[^ ^53^ ^]^ The projector‐augmented wave (PAW) potential sets recommended by the VASP were used. The kinetic‐energy cutoff was set to 500 eV. An 8 × 8 × 12 k‐mesh was taken and the revised PBE for solids (PBEsol) was used for the exchange‐correlation functional.^[^ ^54^ ^]^ The *ε*‐iron carbide structure was created by extending the FeC with NiAs type structure by a factor of 2√3 × 2√3 in the in‐plane direction and making it half deficient of C so that the density of C in the in‐plane direction was constant. The lattice constants and internal coordinates of *ε*‐ iron carbide were experimental values. Calculations were performed for both nonmagnetic and ferromagnetic states and the top of the occupied band at the Γ point in the nonmagnetic phase. The structure was visualized by VESTA.^[^ ^55^ ^]^

## Conflict of Interest

The authors declare no conflict of interest.

## Supporting information



Supporting Information

## Data Availability

The data that support the findings of this study are available on request from the corresponding author. The data are not publicly available due to privacy or ethical restrictions.
