# Supplementary material for: One‐Step Low‐Temperature Synthesis of Metastable ε‐Iron Carbide Nanoparticles with Unique Catalytic Properties Beyond Conventional Iron Catalysts
Source: Small. 2025 Apr 24;21(27):2412217. doi: 10.1002/smll.202412217 (PMC12243695; doi:10.1002/smll.202412217)
Supplement: Supplementary file 1 — Supporting Information [file SMLL-21-2412217-s001.docx]

Supporting Information

One-step Low-Temperature Synthesis of Metastable *ε*-Iron Carbide Nanoparticles with Unique Catalytic Properties Beyond Conventional Iron Catalysts

Yuma Hirayama, Akira Miura, Motoaki Hirayama , Hiroyuki Nakamura, Koji Fujita,
Hiroshi Kageyama, Sho Yamaguchi, Tomoo Mizugaki, and Takato Mitsudome*

**Table of Contents**

1. Characterization

2. Product identification

3. Supplementary reference

1.
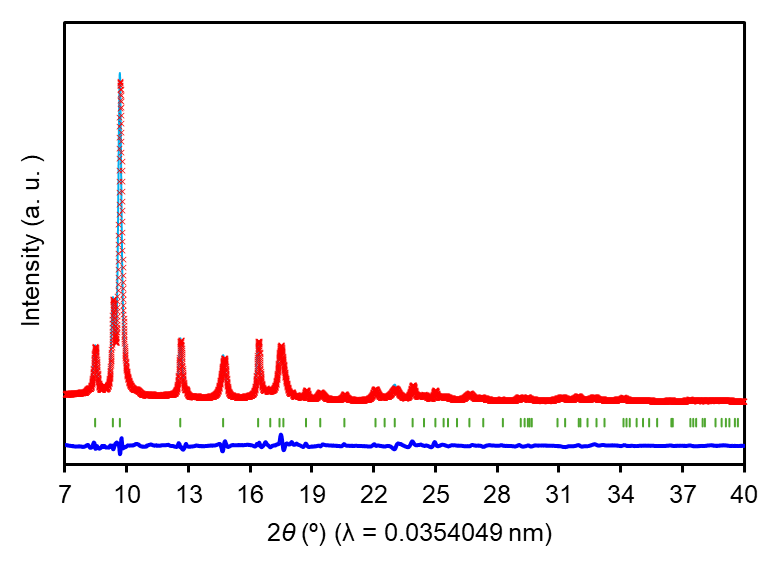
**Characterization**

**Figure S1.** Rietveld refinement profile of synchrotron XRD spectrum. Observed (red), calculated (sky-blue) patterns, and the difference (blue) between them resulting from Rietveld analysis. Green vertical bars denote diffraction position of *ε*-iron carbide.

**
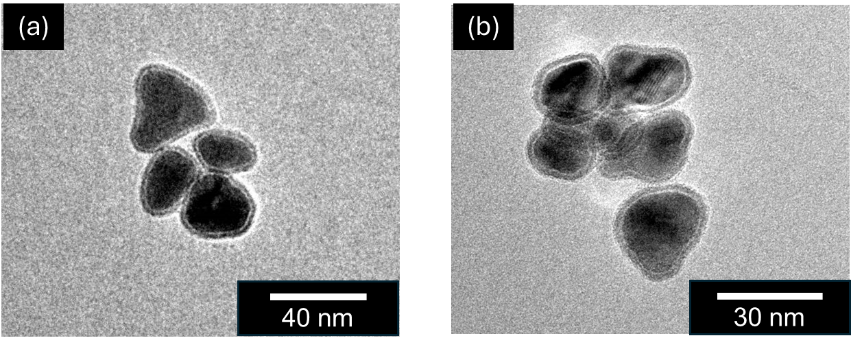
**

**Figure S2.** TEM images of *ε*-iron carbide NPs. The sample formed a core/shell structure due to oxidation upon exposure to air.^[S1]^


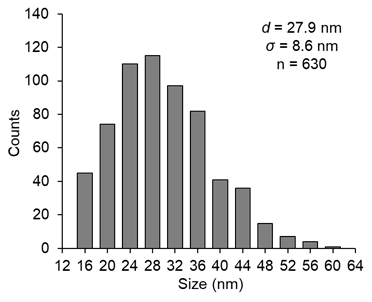


**Figure S3.** Size distribution histogram of *ε*-iron carbide NPs.


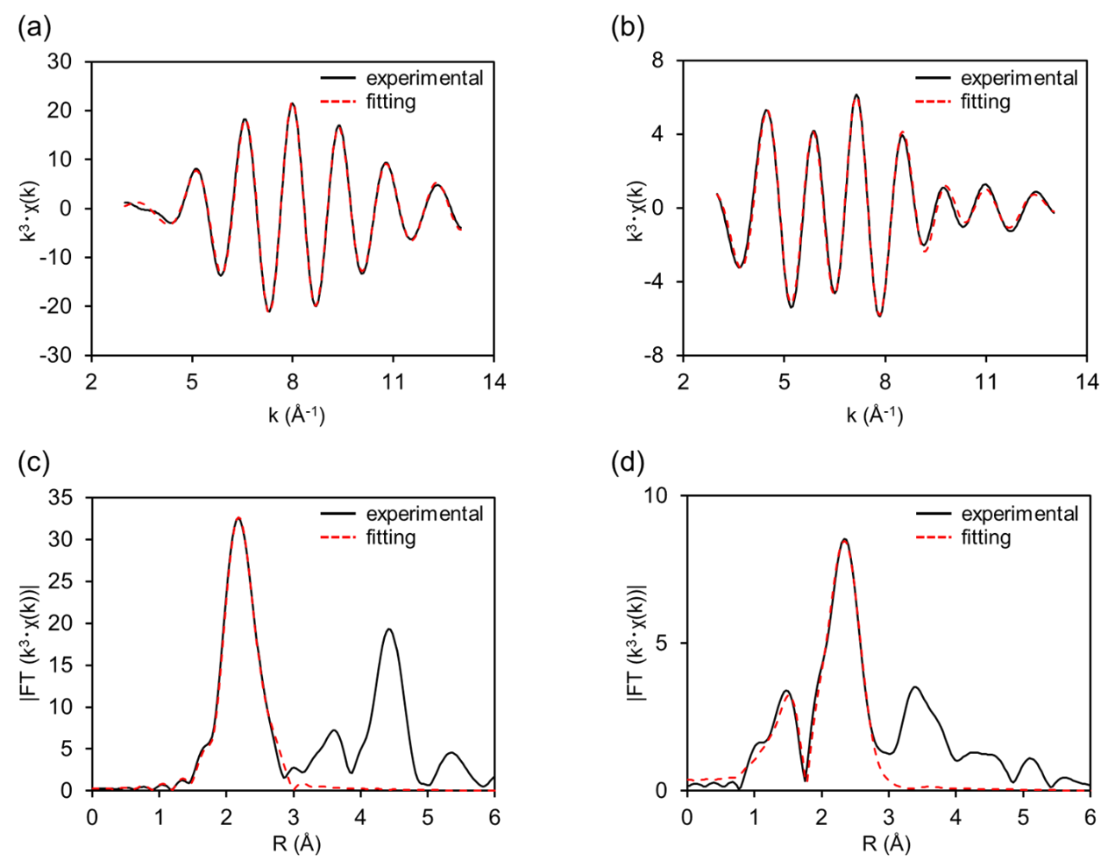


**Figure S4.** Extended X-ray absorption fine structure (EXAFS) fitting curves of Fe foil and *ε*-iron carbide in (a-b) *k*-space and (c-d) *R*-space.


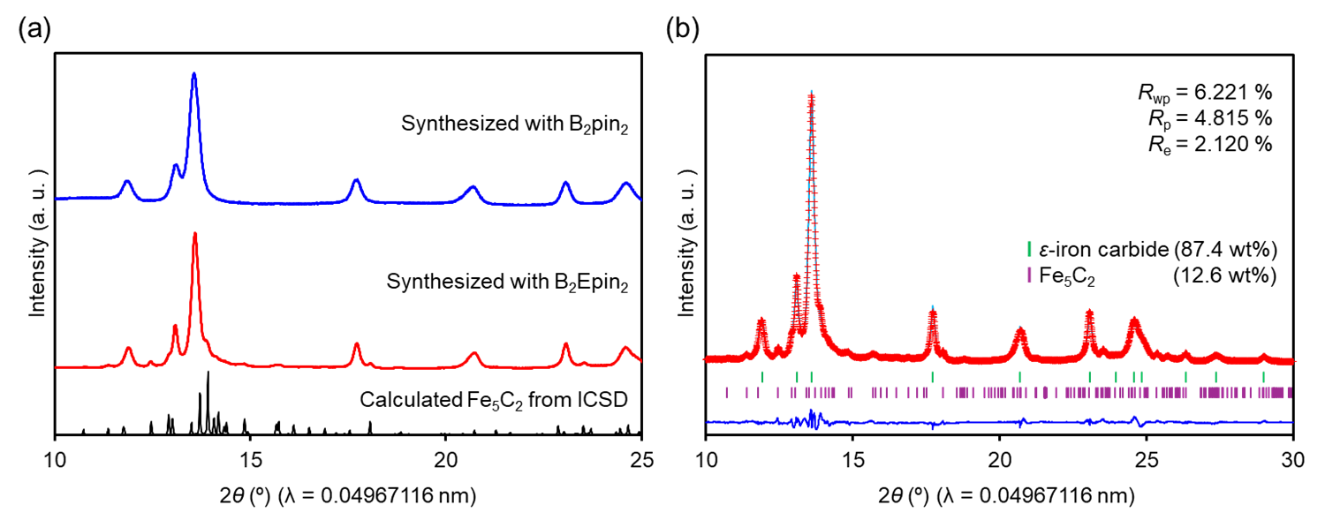


**Figure S5.** (a) Synchrotron XRD spectra of iron carbide synthesized with B_2_pin_2_ and B_2_Epin_2_ and (b) Rietveld refinement profile of synchrotron XRD spectrum of iron carbide synthesized with B_2_Epin_2_. Observed (red), calculated (sky-blue) patterns, and the difference (blue) between them resulting from Rietveld analysis. Green and purple vertical bars denote positions of Bragg diffractions of *ε*-iron carbide and Fe₅C₂ (ICSD CollCode 423885).

**
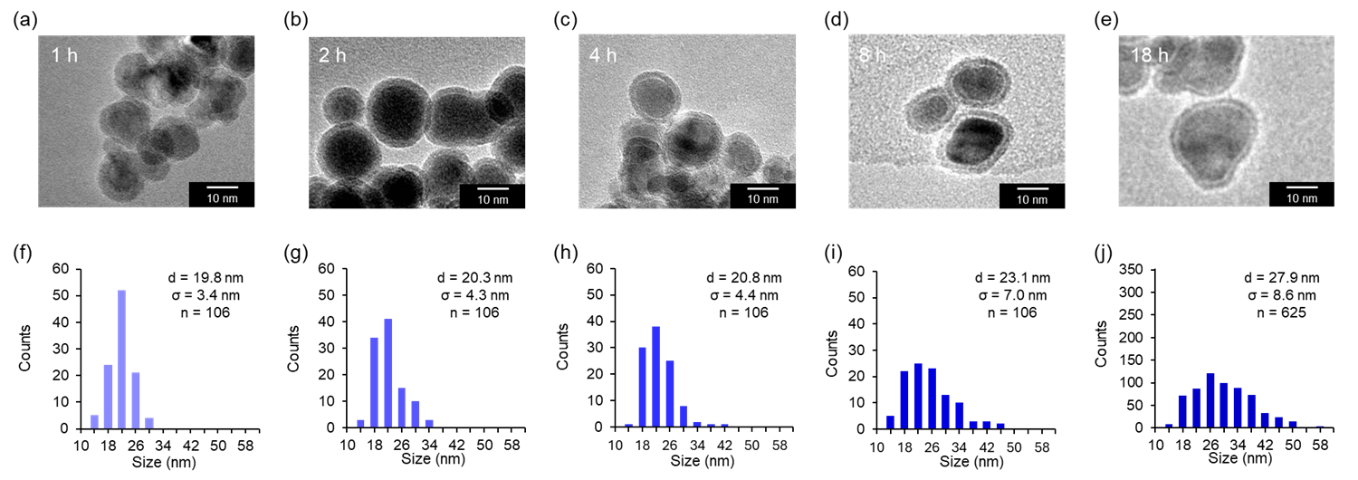
Figure S6.** Structural evolution of *ε*-iron carbide NPs. TEM images and size distribution histogram of sample obtained after 1 h (a and f), 2 h (b and g), 4 h (c and h), 8 h (d and i), 18 h (e and j).


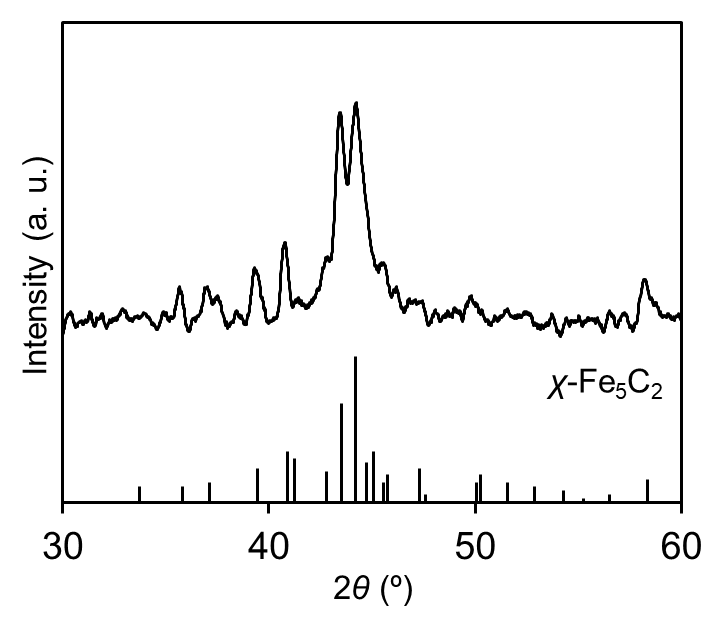


**Figure S7.** XRD pattern of *χ*-Fe_5_C_2_. (black bars below the pattern show the diffraction peaks reffering to ICSD CollCode 423885).


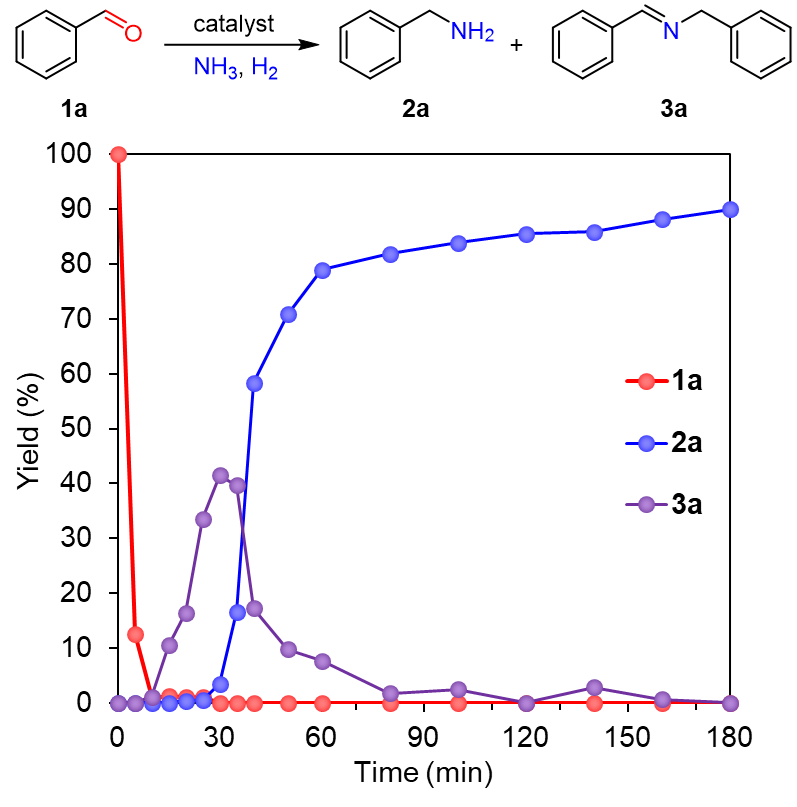


**Figure S8.** Time-course of the *ε*-iron carbide/SiO_2_ catalyst system for the reductive amination of **1a**. Reaction conditions: *ε*-iron carbide/SiO_2_ (Fe: 10 mol%), **1a** (0.5 mmol), ethanol (3 mL), NH_3_ (0.3 MPa), H_2_ (3.7 MPa), 150 ℃.


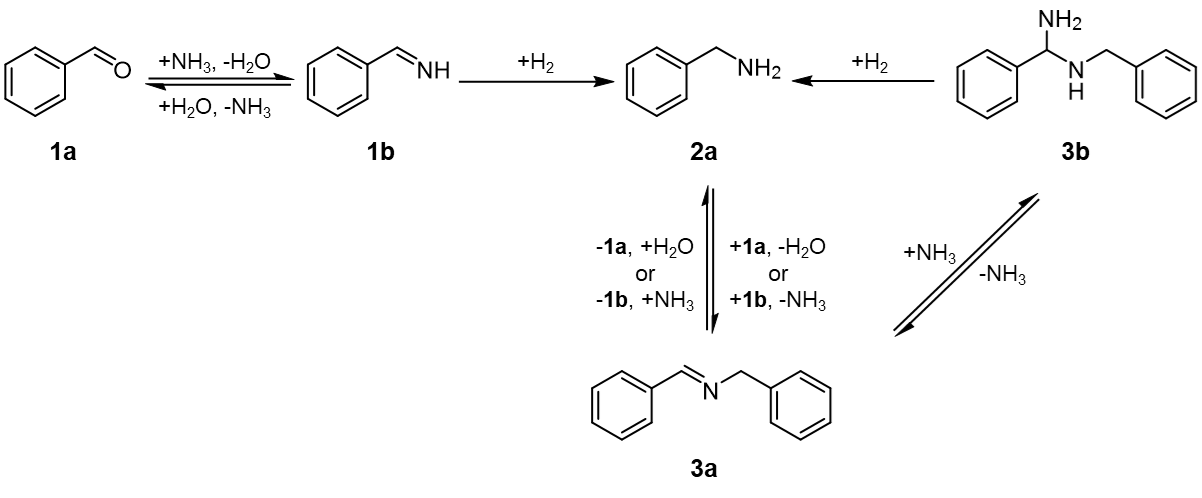


**Figure S9.** A plausible reaction pathway for reductive amination of **1a** to **2a**.

In the initial stage, **1a** reacts with NH₃ to form benzylideneamine (**1b**), which is subsequently hydrogenated to yield **2a**. At the later stage, **2a** can react with either **1a** or **1b** to form **3a**. This intermediate **3a**, upon reacting with NH₃, can provide **2a** via two routes: (i) **3a** produces the unstable geminal diamine (**3b**), which undergoes hydrogenolysis to **2a**, or (ii) it decomposes to regenerate imine (**1b**), which is subsequently hydrogenated to **2a**.


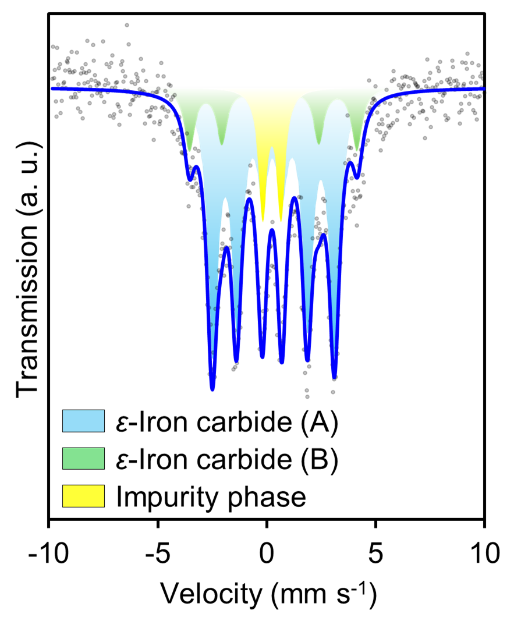


**Figure S10.** ^57^Fe Mössbauer spectrum of used *ε*-iron carbide NPs.

**
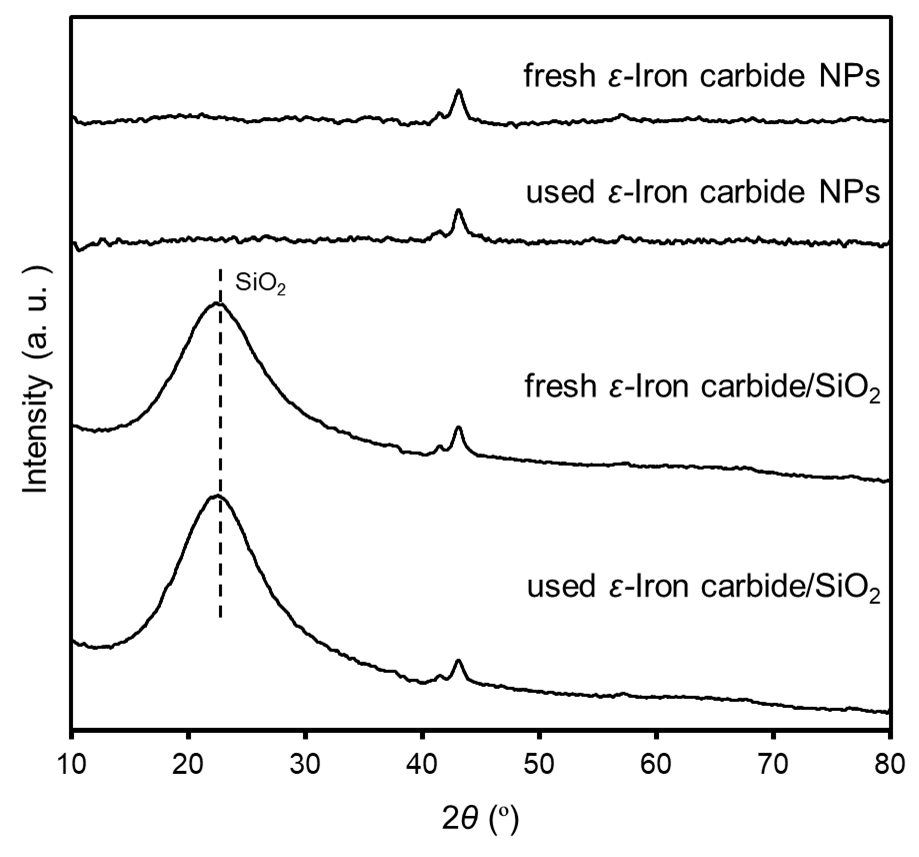
**

**Figure S11.** XRD patterns of fresh *ε*-iron carbide NPs, used *ε*-iron carbide NPs, fresh *ε*-iron carbide/SiO_2_, and used *ε*-iron carbide/SiO_2_.


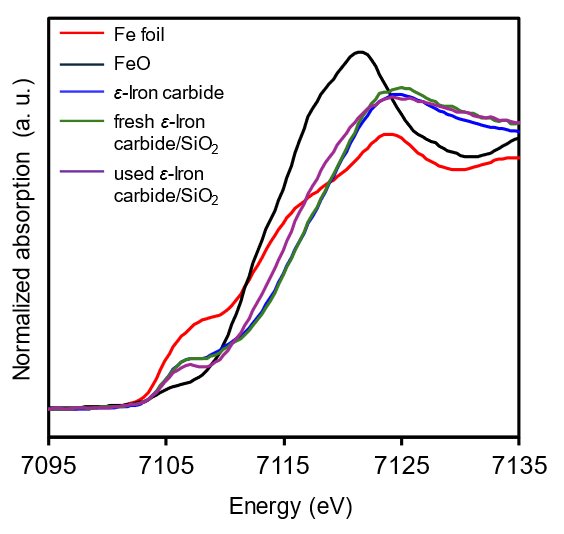


**Figure S12.** Fe *K*-edge XANES spectra of Fe foil, FeO, and *ε*-iron carbide, fresh *ε*–iron carbide/SiO_2_ and used *ε*-iron carbide/SiO_2_.


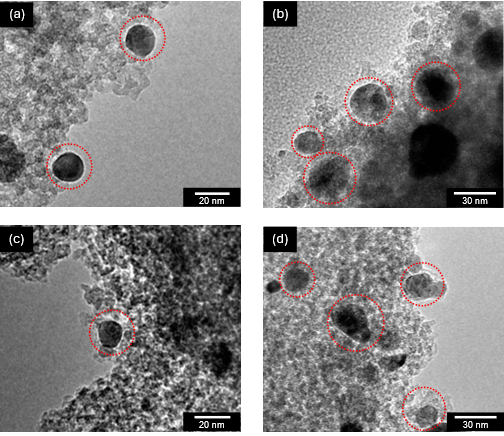


**Figure S13.** TEM images of (a) fresh and (b) used *ε*-iron carbide/SiO_2_.

**Table S1.** Experimental conditions and analytical results.

| Compound | *ε*-iron carbide |
| --- | --- |
| Chemical formula | C_0.48278_Fe |
| *Mr* | 61.644 |
| Crystal system | Hexagonal |
| Space group | *P*6_3_/*mmc* (No. 194) |
| Hall symbol | –*P* 6c 2c |
| Center of symmetry | An inversion center of the origin |
| a/Å | 2.77005(9) |
| b/Å | 2.77005(9) |
| c/Å | 4.3557(2) |
| α/° | 90 |
| β/° | 90 |
| γ/° | 120 |
| *V*/ Å^3^ | 28.944(2) |
| *Z* | 2 |
| *D_x_/*Mg m^-3^ | 7.073 |
| *F*(000) | 57.79 |
| Radiation source | Synchrotron radiation |
| Wavelength, λ/ Å | 0.354049 |
| Goniometer | Debye–Scherrer geometry |
| 2*θ*_min_/° | 7.000 |
| 2*θ*_max_/° | 40.000 |
| Δ2*θ*/° | 0.005 |
| Data points | 13910 |
| dmin/ Å | 0.51759 |
| *R*_wp_ | 0.05875 |
| *R*_e_ | 0.04957 |
| *S* = *R*_wp_/*R*_e_ | 1.1853 |
| *R*_p_ | 0.04392 |
| *R*_B_ | 0.02878 |
| *R*_F_^2^ | 0.01673 |
| *R*_F_ | 0.01697 |

**Table S2.** Fractional coordinates, isotropic atomic displacement parameters, and occupancies.mW denotes an integrated combination of the multiplicity and Wyckoff letter.

| Site | *mW* | *x* | *y* | *z* | *U/* Å^2^ | *g* |
| --- | --- | --- | --- | --- | --- | --- |
| Fe | 2*c* | 1/3 | 2/3 | 1/4 | 0.00456(6) | 1 |
| C | 2*a* | 0 | 0 | 0 | 0.017(2) | 0.482(9) |

| Sample | IS^a^ (mm s^−1^) | QS^b^ (mm s^−1^) | *H*_hf_^c^ (T) | Γ (mm s^−1^) | Area (%) | Phase |
| --- | --- | --- | --- | --- | --- | --- |
| fresh  *ε*-iron carbide | 0.21 ± 0.01 | 0.07 ± 0.02 | 17.4 ± 0.1 | 0.67 ± 0.03 | 72 | *ε*-iron carbide (A) |
|  | 0.20 ± 0.05 | 0.0 ± 0.1 | 24.3 ± 0.4 | 0.67 ± 0.03 | 14 | *ε*-iron carbide (B) |
|  | 0.24 ± 0.02 | 0.90 ± 0.03 | - | 0.48 ± 0.06 | 14 | impurity phase |
| used  *ε*-iron carbide | 0.26 ± 0.01 | 0.07 ± 0.02 | 17.4 ± 0.1 | 0.59 ± 0.03 | 70 | *ε*-iron carbide (A) |
|  | 0.23 ± 0.04 | 0.13 ± 0.08 | 23.9 ± 0.3 | 0.59 ± 0.03 | 17 | *ε*-iron carbide (B) |
|  | 0.24 ± 0.03 | 0.89 ± 0.05 | - | 0.49 ± 0.08 | 13 | impurity phase |

**Table S3.** Fitting of ^57^Fe Mössbauer spectrum of fresh and used *ε*-iron carbide.

^a^Isomer shift. ^b^Quadrupole splitting. ^c^Hyperfine field.

**Table S4.** Curve fitting of Fe *K*-edge EXAFS spectra for Fe foil and *ε*-iron carbide.

| Sample | Shell | *CN*^a^ | *R*^b^ (Å) | D.W.^c^ | R factor (%) |
| --- | --- | --- | --- | --- | --- |
| Fe foil | Fe–Fe | 8 | 2.484 ± 0.002 | 0.0053 ± 0.0006 | 5.8 |
|  |  | 6 | 2.865 ± 0.005 | 0.008 ± 0.001 |  |
| *ε-*iron carbide | Fe–C | 3.0 ± 0.9 | 1.967 ± 0.01 | 0.007 ± 0.002 | 9.6 |
|  | Fe–Fe | 9.9 ± 0.9 | 2.737 ± 0.006 | 0.015 ± 0.001 |  |

^a^Coordination number. ^b^Bond distance. ^c^Debye-Waller factor.

1. **Product identification**

All reaction products were characterized by GC and NMR spectroscopy. The ^1^H and ^13^C NMR chemical shifts of the products agreed with those of previously reported values.

benzylamine hydrochloride [S2]

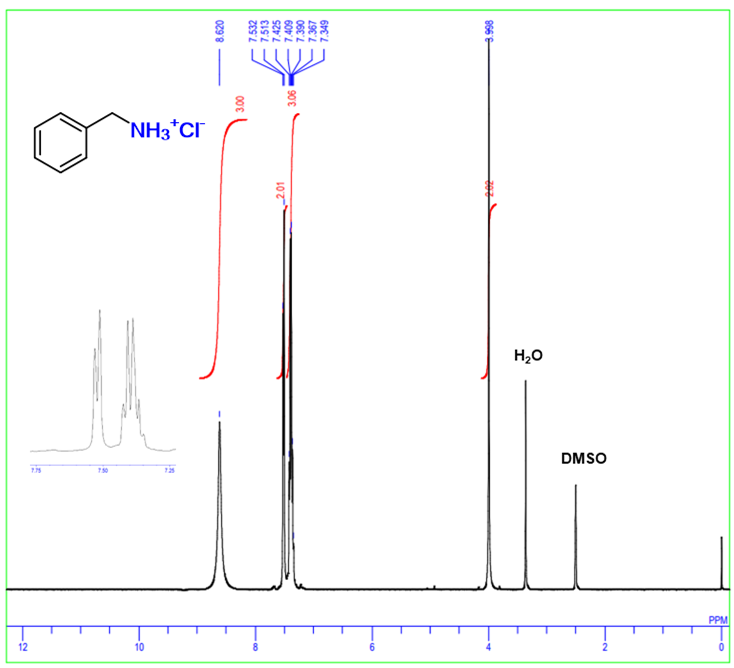
^1^H NMR (DMSO-*d_6_,* 400 MHz): δ = 8.62 (br s, 3H), 7.52 (d, *J* = 7.3 Hz, 2H), 7.43–7.35 (m, 3H), 4.00 (s, 2H); ^13^C NMR (DMSO-*d_6_,* 100 MHz): δ = 134.1, 129.0, 128.5, 128.3, 42.1


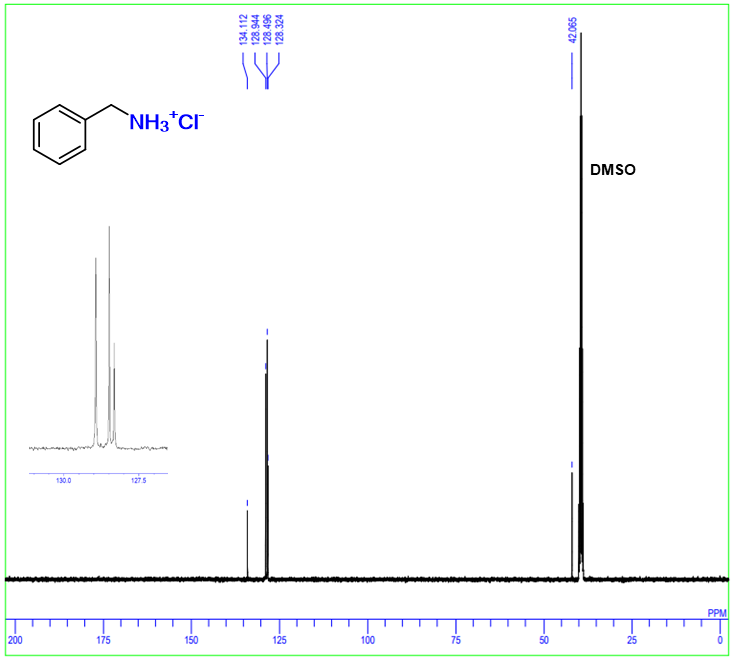
^1^H NMR spectrum of benzylamine hydrochloride

^13^C NMR spectrum of benzylamine hydrochloride

1. **Supplementary Reference**

[S1] S. Janbroers, J. N. Louwen, H. W. Zandbergen, P. J. Kooyman, *J. Catal.* **2009**, *268*, 235.

[S2] F. Chen, C. Topf, J. Radnik, C. Kreyenschulte, H. Lund, M. Schneider, A. E. Surkus, L. He, K. Junge, M. Beller, *J. Am. Chem. Soc.* **2016**, *138*, 8781.
